# Paracrine Mechanisms of *Histoplasma*-Infected BM-MSCs Alter Signaling, Proliferation, and Viability of HSPCs

**DOI:** 10.3390/jof12100723

**Published:** 2026-09-25

**Authors:** Carolina Rodríguez-Echeverri, Brajhan Torres, Rafael A. Ramírez-Trujillo, Beatriz L Gómez, Ángel González

**Affiliations:** 1 Basic and Applied Microbiology Group (MICROBA), School of Microbiology, Universidad de Antioquia, Calle 67 No. 53–108, Office: 5–103, Medellín 050010, Colombia; carolina.rodrigueze@udea.edu.co (C.R.-E.); brajhan.torres@udea.edu.co (B.T.); rafael.ramirezt@udea.edu.co (R.A.R.-T.); 2 Translational Microbiology and Emerging Diseases Research Group (MICROS), School of Medicine and Health Sciences, Universidad del Rosario, Bogotá 111221, Colombia; beatriz.gomez@urosario.edu.co

**Keywords:** mesenchymal stromal cells, hematopoietic stem and progenitor cells, *Histoplasma capsulatum*, paracrine signaling

## Abstract

Stem cells possess regenerative and immunomodulatory capabilities; however, their role can be paradoxical during infections that alter hematopoiesis. Disseminated histoplasmosis is clinically associated with anemia and pancytopenia. Previous studies have shown that *Histoplasma capsulatum* can internalize and alter the function of mesenchymal stromal cells (MSCs) and hematopoietic stem cells and their progenitors (HSPCs). This study aimed to determine whether infection of BM-MSCs with *H. capsulatum* modulates HSPC viability, proliferation, and signaling through paracrine mechanisms. MSCs and HSPCs were isolated from the bone marrow of C57BL/6 mice. MSCs were infected with *H. capsulatum* yeast and subsequently co-cultured with HSPCs using transwell plates; additionally, HSPCs were treated with supernatants from infected MSCs. HSPCs were analyzed by flow cytometry to assess apoptosis, necrosis, and proliferation. Relative expression of genes related to Notch signaling was quantified by RT-qPCR. HSPCs cultured with conditioned medium or *H. capsulatum*-infected MSCs showed significantly increased apoptosis and necrosis, accompanied by reduced proliferation. Additionally, *NOTCH1*, *NOTCH2*, and *JAGGED1* expression increased, whereas *BCL2* expression decreased. These findings indicate that *H. capsulatum*-infected MSCs alter signaling pathways and cell fate in HSPCs through a paracrine mechanism.

## 1. Introduction

Systemic infections are characterized by inducing stress in the bone marrow, which is reflected in increased or decreased leukocyte and platelet counts in peripheral blood, a process known as emergency hematopoiesis. Patients with chronic inflammatory conditions caused by infections or autoimmune diseases frequently present with anemia, thrombocytopenia, leukopenia, or pancytopenia [1,2,3,4,5]. Emergency hematopoiesis is a process regulated either by the direct interaction between bone marrow cells and invading pathogens or indirectly through infected or activated cells that secrete cytokines in response to infection [6].

Several experimental studies have demonstrated that infection by different microorganisms can induce the expansion of hematopoietic stem and progenitor cells (HSPCs) in response to increased demand. Conversely, infection may also lead to HSPC depletion through exhaustion of cellular reserves via mechanisms such as apoptosis or necrosis [6,7,8]. Additionally, other studies have reported that certain infectious agents induce changes in the expression of surface markers on HSPCs, either through direct effects or indirectly via cytokine induction in these and other bone marrow cells, including mesenchymal stromal cells (MSCs) [6,9,10,11]; thus, alterations in MSC function during infection may affect the balance between HSPC maintenance, proliferation, and cell death. In this context, it has been documented that both HSPCs and MSCs express receptors such as *Toll*-like receptors (TLRs), among others, which enable them to recognize pathogens through surface-associated molecular patterns [12,13,14,15]. Activation of these receptors can initiate intracellular signaling pathways, leading to changes in the expression of inflammatory mediators and genes involved in cell survival, proliferation, and differentiation. Moreover, pathways regulating cell fate and survival, including the Bcl-2 family and Notch signaling, contribute to the maintenance of hematopoietic homeostasis. In particular, the balance between pro- and anti-apoptotic Bcl-2 family members regulates the intrinsic apoptotic pathway, whereas Notch signaling contributes to the maintenance and differentiation of HSPCs [16,17]. Together, these molecular mechanisms provide a basis through which alterations in the bone marrow microenvironment may influence hematopoiesis during infection [18,19].

Recent studies from our group have demonstrated that *Histoplasma capsulatum*, the etiological agent of histoplasmosis, can be internalized by both HSPCs and MSCs and activate these cells through mechanisms dependent on pattern recognition receptors, including TLR2, TLR4, and Dectin-1. This interaction induces differential expression of TLRs and Dectin-1 in both cell types, as well as genes encoding pro-inflammatory cytokines such as IL-1β, IL-6, IL-17, TNF-α, and TGF-β. Furthermore, it promotes apoptosis and necrosis and alters the differentiation of MSCs into mesodermal lineages [20].

In the present study, we evaluated whether infection of MSCs with *Histoplasma capsulatum* alters their paracrine capacity to regulate the proliferation and survival of HSPCs. To this end, we employed an in vitro model consisting of MSC and HSPC co-cultures and conditioned media derived from infected MSCs to culture HSPCs, followed by analysis using flow cytometry and molecular techniques. This research aims to determine whether the pancytopenia and anemia characteristic of disseminated histoplasmosis result from an indirect or paracrine modulation of the hematopoietic niche triggered by the stromal cell response to the fungal pathogen.

## 2. Materials and Methods

### 2.1. Isolation, Purification, and Culture of Hematopoietic Stem Cells and Their Progenitors (HSPCs)

Bone marrow cells were obtained from the femur and tibia of 8-week-old C57BL/6 mice. HSPCs [Lin-c-Kit+ Sca-1+ (LKS)] were purified from bone marrow using a commercial kit with monoclonal antibodies coupled to magnetic microbeads (Miltenyi Biotec, Bergisch Gladbach, Germany) in a two-step process following the manufacturer’s instructions. Initially, cells positive for the following antigens were depleted: CD45, CD45R (B220), CD11b, CD5, Gr1 (Ly-6/C), 7-4, and Ter-119. Subsequently, the cells were purified using an anti-Sca-1 antibody coupled to magnetic microbeads. Purified cells were cultured in RPMI-1640 medium supplemented with 2 mM L-glutamine, 5% inactivated fetal bovine serum (FBS) (GIBCO), 1% penicillin-streptomycin (GIBCO), and stem cell factor (SCF) (20 ng/mL), to promote cell viability.

### 2.2. Isolation, Purification, and Culture of Bone Marrow-Derived Mesenchymal Stromal Cells (BM-MSCs)

Protocols for MSC isolation and purification were adapted from a previous report by Rodríguez-Echeverri et al. [20]. Briefly, BM-MSCs were purified using magnetic cell depletion. After 48 h, non-adherent cells were removed, and adherent cells were cultured in low-glucose DMEM for 7 days. Characterization was confirmed by inverted light microscopy through observation of the characteristic fibroblastic morphology and uniform monolayer growth.

Multipotency was determined using the protocol described by Rodriguez-Echeverri et al. [20]. BM-MSCs were induced to differentiate into osteogenic, adipogenic, and chondrogenic lineages under standard culture conditions, using the commercial StemPro kit (Thermo Fisher Scientific, Waltham, MA, USA). Cells were seeded at appropriate densities, depending on the lineage, and cultured for 20 days with medium replacement every 3–4 days. Following induction, cells were fixed with 4% formaldehyde and subjected to immunofluorescence staining using lineage-specific markers, including osteocalcin for osteogenic differentiation, fatty acid-binding protein 4 (FABP4) for adipogenic differentiation, and aggrecan for chondrogenic differentiation. Detection was performed using DyLight™ 594-conjugated secondary antibodies (Termo Fisher Scientific (Invitrogen), Waltham, MA, USA), and nuclei were counterstained with Hoechst.

Immunophenotyping was confirmed by flow cytometry (FACS Canto II system, BD Biosciences, San Jose, CA, USA) using specific fluorochrome-labelled monoclonal antibodies: anti-CD45 conjugated with fluorescein isothiocyanate (FITC), anti-SCA-1 conjugated with Pacific Blue, anti-CD44 conjugated with phycoerythrin (PE)-Cy5, anti-CD73 conjugated with R-phycoerythrin (PE), and anti-CD105 conjugated with allophycocyanin (APC) (BD Pharmingen, San Diego, CA, USA).

### 2.3. Histoplasma capsulatum Yeasts

*Histoplasma capsulatum* strain G217B was used in this study. The fungus was cultured in the yeast phase using Brain-Heart Infusion (BHI) liquid medium (HiMedia Laboratories Pvt. Ltd., Mumbai, India), supplemented with 1% glucose (Sigma-Aldrich, St. Louis, MO, USA), and 100 U/mL Penicillin–100 μg/mL Streptomycin (GIBCO Invitrogen Corporation, Carlsbad, CA, USA). Cultures were incubated at 36 °C with constant agitation at 150 rpm for 48 h. Before experimental use, yeast cells were centrifuged at 3000 rpm for 5 min, resuspended in phosphate-buffered saline (PBS), and quantified using a hemocytometer.

### 2.4. Infection of BM-MSCs with H. capsulatum Yeasts and Co-Cultures with HPSCs

BM-MSCs were infected with *H. capsulatum* yeast at an MOI of 5 for 72 h. Following infection, the supernatant was collected and used as conditioned media (CM). In other experiments, the BM-MSC monolayer was washed with DMEM media, and then 0.5 mL containing 2 × 10^5^ HSPCs was added to the medium described above using a Transwell membrane. After 24 h of co-culture, non-adherent cells, corresponding to HPSCs, were collected. HSPCs were resuspended in the conditioned medium of BM-MSCs previously infected or non-infected with the fungus, maintaining a suspension of 2 × 10^5^ HSPCs in 0.5 mL of conditioned medium and cultured for 24 h.

In summary, four experimental groups were established: (i) co-cultures of uninfected-MSC + HSPC, (ii) co-culture of *Histoplasma*-infected-MSC + HSPC, (iii) HSPC + CM from uninfected BM-MSCs, and (iv) HSPC + CM from *Histoplasma*-infected BM-MSCs.

### 2.5. Cell Proliferation Assays

For cell proliferation assays, HSPCs were labeled with 10 µM 5-bromo-2-deoxyuridine (BrdU) in culture medium for 90 min, then co-cultured with BM-MSCs or CM in low-glucose DMEM for 24 h, and using a commercial BrdU kit (BD, Pharmingen, San Diego, CA, USA) according to the manufacturer’s instructions [12], the HSPCs were subsequently analyzed by flow cytometry.

### 2.6. Apoptosis Assays

To determine apoptosis by flow cytometry, the commercial ApoDetect kit (Invitrogen Corporation, Carlsbad, CA, USA) was used. HSPCs from the different groups were treated with Annexin V coupled to FITC and with propidium iodide, according to the manufacturer’s instructions.

### 2.7. Analysis of the Expression of Genes Associated with HSPC Cell Signaling

Total RNA was obtained from HSPCs using TRIzol reagent (Invitrogen, Carlsbad, CA, USA). The sample was also processed using DNase I treatment (Thermo Fisher Scientific Inc., Waltham, MA, USA). Complementary DNA (cDNA) was synthesized from 500 ng of RNA using a commercial RT-qPCR synthesis kit (Solis Biodyne, Tartu, Estonia) according to the manufacturer’s instructions. qPCR was performed using the Maxima SYBR Green/Fluorescein qPCR Master Mix (2X) kit according to the manufacturer’s instructions (Thermo Fisher Scientific Inc., Waltham, MA, USA).

Validation of the constitutive or “housekeeping” gene used for normalizing mRNA expression was performed according to the methodology previously reported by our group [20]. Changes in the mRNA expression of the target genes were quantified relative to the expression of the constitutive gene encoding the enzyme glyceraldehyde-3-phosphate dehydrogenase (GAPDH). The real-time PCR detection system used to measure gene expression levels was the CFX96 (Bio-Rad, Hercules, CA, USA).

The relative expression of the *NOTCH1*, *NOTCH2*, *JAGGED1*, and *JAGGED2* genes (components of the Notch signaling pathway) was assessed, along with the *BCL-2* gene, which acts as an anti-apoptotic protein. Specific primers were used to determine the relative expression of genes encoding for these signaling molecules (Table 1).

### 2.8. Ethical Considerations

This study was conducted in accordance with the regulations outlined in established by Colombian Law 84/1989 and Resolution No. 8430/1993, as well as with the guidelines issued by the European Union and the Canadian Council on Animal Care. The protocol was approved by the Ethics Committee for Experimentation with Animals of the Universidad de Antioquia (Act No. 151).

### 2.9. Statistical Analysis

All data were analyzed using Prisma GraphPad Software (version 8.0). Statistical analyses were performed using tests such as the Mann–Whitney U test or Student’s *t*-test for comparisons between two groups. In all cases, a *p*-value < 0.05 was considered statistically significant.

## 3. Results

### 3.1. Characterization of the BM-MSCs

The identification of the BM-MSCs was carried out from a primary culture. After seeding, a cell population with fibroblastic morphology and uniform monolayer growth was observed. Subsequently, transformation into specific mesodermal lineages was successfully induced, confirming their multipotent capacity (Figure 1). The phenotypic profile showed a positive population of cells for the mesenchymal markers CD44, CD105, and CD73, and negative for CD45 (Figure 2).

### 3.2. BM-MSCs Infected with H. capsulatum Increase Apoptosis and Necrosis of HSPC by a Paracrine Mechanism

Apoptosis was shown to have increased significantly in HSPCs co-cultured with *H. capsulatum*-infected BM-MSCs, as well as in HSPCs cultured in conditioned medium derived from infected MSCs, compared with their respective controls (Figure 3A). Regarding necrosis, only those HSPCs cultured with conditioned medium from infected MSCs showed a marked increase in comparison with the other groups (Figure 3B). These findings indicate that exposure to factors released by infected MSCs is associated with increased HSPC cell death.

### 3.3. Conditioned Media from BM-MSCs Infected with Hc Alters HSPCs Proliferation

HSPC proliferation was assessed by BrdU incorporation using flow cytometry. Proliferation was significantly increased in HSPCs exposed to *H. capsulatum* and in those cultured with conditioned medium from uninfected MSCs. In contrast, proliferation was significantly reduced in HSPCs co-cultured with uninfected MSCs and in those exposed to conditioned medium from *H. capsulatum*-infected MSCs (Figure 4).

### 3.4. MSCs Infected with Histoplasma Alter the Expression of Signaling Genes in HSPCs Through a Paracrine Mechanism

The relative expression of genes associated with cell signaling was evaluated in HSPCs under different culture conditions. *NOTCH1* expression was significantly increased in HSPCs under the following culture conditions: infection with *Histoplasma*, co-cultured with infected or uninfected MSCs, and with conditioned medium from MSCs infected with the fungus (Figure 5A), indicating that soluble factors derived from MSCs could modulate Notch1 signaling in HSPCs. Regarding the relative expressions of *NOTCH2* and *JAGGED1*, we observed that both genes showed a similar trend, with a significantly higher expression in those HSPCs co-cultured with either uninfected or infected MSCs (Figure 5B,C). In the case of the *JAGGED2* gene expression, no significant differences in gene expression were observed among treatments or interactions with MSC cells (Figure 5D).

### 3.5. Histoplasma Infection Modulates BCL-2 Expression in HSPCs

We observed higher *BCL2* expression in HSPCs co-cultured with uninfected MSCs. Interestingly, when these hematopoietic progenitor cells were co-cultured with infected-MSCs or cultured in the presence of conditioned media from infected or non-infected MSCs, *BCL2* expression decreased (Figure 6). These findings suggest that soluble factors derived from MSCs could contribute to the regulation of anti-apoptotic signaling in HSPCs.

## 4. Discussion

Systemic mycoses represent a growing global health challenge, a reality recently underscored by the World Health Organization (WHO), which classified *Histoplasma* spp. within the High-Priority group of its landmark Fungal Priority Pathogens List [21]. Although *Histoplasma capsulatum* is classically defined as a thermally dimorphic fungus restricted to specific geographic regions, its study holds broad biological and clinical significance that extends far beyond regional endemicity [22]. Amid expanding populations of immunocompromised hosts and growing global interconnectivity, systemic mycoses represent an increasingly critical challenge to public health. Notably, patients with underlying immunological alterations can develop dissemination of histoplasmosis to the bone marrow, manifesting clinically as anemia and pancytopenia [23], hematological findings that reflect significant compromise of the progenitor cell compartment within the bone marrow niche. Within this framework, the findings of the present study demonstrate that *H. capsulatum* infection of bone marrow mesenchymal stromal cells (BM-MSCs) disrupts the hematopoietic niche.

The concept of the hematopoietic niche, originally proposed by Schofield et al. [24], suggests that the maintenance of hematopoietic stem and progenitor cells depends on local signals generated by specialized neighboring cells [24]. In this context, among the various cellular components of the bone marrow niche, mesenchymal stromal cells represent a key functional element, as they regulate HSPC quiescence, survival, proliferation, and differentiation through direct cell–cell interactions, extracellular matrix components, and paracrine mechanisms. These mechanisms involve the secretion of a wide range of soluble factors collectively known as the secretome [25,26]. This regulatory capacity is associated with the expression of diverse cytokine and chemokine receptors on HSPCs, enabling them to sense microenvironmental signals and respond indirectly to infection [6,11]. Based on these observations, the present study evaluated the paracrine modulation exerted by *H. capsulatum*-infected MSCs on HSPC signaling, proliferation, and viability.

The findings of this study indicate that infection of MSCs with *H. capsulatum* alters HSPC viability, proliferation, and signaling through a paracrine mechanism. The observed changes in HSPCs under both co-culture conditions and exposure to conditioned medium suggest that, in addition to the direct effects of the fungus on HSPCs [12], infection also impairs the supportive function of the stromal niche. This effect may be associated with previously reported alterations in the expression of cytokine-encoding genes in infected MSCs [20]. To support these findings, MSCs were thoroughly characterized based on their fibroblast-like morphology, their capacity for osteogenic, adipogenic, and chondrogenic differentiation, and their immunophenotypic profile (CD44^+^, CD73^+^, CD105^+^, and CD45^−^), in accordance with the criteria established by the International Society for Cell and Gene Therapy (ISCT) [27], thereby ensuring the validity of the experimental model.

On the other hand, several studies have shown that cytokines regulate HSC function through multiple mechanisms, including modulation of proliferation, induction of lineage-specific differentiation, and activation of apoptotic pathways. Additionally, microbial components recognized by TLRs can trigger cell death processes, including apoptosis and necroptosis [6,28,29,30]. Consistent with this notion, the increased apoptosis observed in this study suggests that MSCs differentially alters their capacity to regulate apoptotic processes depending on their interaction with HSPCs. This finding supports a paracrine mechanism of regulation, which may enhance pro-apoptotic signaling or reduce anti-apoptotic support in HSPCs [31,32]. Our observations align with the findings of Muddineni et al., who established that mesenchymal stromal cells exert a protective regulatory role by inhibiting ionizing radiation-induced apoptosis in hematopoietic stem and progenitor populations [33].

This effect on apoptosis correlates with the relative expression of the *BCL-2* gene, which was found to be higher in HSPCs co-cultured with uninfected MSCs. These findings suggest that when uninfected MSCs communicate in a paracrine fashion with HSPCs, they inhibit HSPC apoptosis by upregulating *BCL-2* expression; however, this effect is less pronounced if the MSCs are infected with the fungus.

The relationship between apoptosis and Bcl-2 is particularly relevant, as apoptotic regulation differs between hematopoietic stem cells and progenitor populations. Bcl-2, a key anti-apoptotic protein involved in the intrinsic apoptotic pathway, is not essential for the survival of murine HSPCs under steady-state conditions [31,34,35]. Although HSPCs maintain a high apoptotic threshold to prevent unnecessary cell death, they also retain active pro-apoptotic mechanisms [31,34,35]. Therefore, the pattern observed in this study suggests a relative loss of anti-apoptotic support provided by uninfected MSCs when these cells become infected.

Additionally, our results regarding necrosis indicate that MSC infection does not significantly alter necrosis levels in HSPCs under co-culture conditions. In contrast, conditioned medium from infected MSCs failed to adequately regulate necrosis, resulting in a marked increase in this process. These findings suggest that paracrine communication is required to maintain proper control of necrotic signaling pathways. Accordingly, the observed differences imply that soluble factors accumulated during MSC infection may induce cellular damage or inflammatory stress, compromising HSPC membrane integrity [7,8,31].

Furthermore, co-culture with MSCs maintained low HSPC proliferation, whereas it increased significantly when using conditioned medium from uninfected MSCs. This finding is consistent with previous reports demonstrating that physical contact within the bone marrow niche provides key regulatory cues, including adhesion-mediated signaling that cannot be fully recapitulated by soluble factors alone [26]. While previous studies have mainly focused on the direct effects of infectious agents or inflammatory mediators on hematopoietic cells [11,13,14], our findings provide evidence that infection of BM-MSCs can also influence HSPC proliferation through the surrounding cellular microenvironment. The contrasting effects observed with conditioned media from uninfected versus infected MSCs further support the contribution of infection-induced changes in the paracrine profile of BM-MSCs. Our results differ from those reported by Crippa et al., who demonstrated that, in human cells, conditioned medium is less efficient than direct co-culture in promoting the proliferation and maintenance of HSPCs [36]. Specifically, the increased proliferation observed in HSPCs cultured with conditioned media from uninfected MSCs, which resembles the response seen in HSPCs directly infected with *H. capsulatum*, suggests that MSCs in co-culture secrete factors that promote a supportive or quiescent state. However, this supportive profile is disrupted following fungal infection, resulting in the reduced proliferation observed with conditioned media from infected MSCs.

These results are better contextualized considering the work by Walenda et al., who demonstrated enhanced HSPC proliferation during direct co-culture with MSCs, underscoring that stromal modulation depends heavily on the nature of the interaction between MSCs and HSPCs [37]. Additionally, other studies have highlighted the critical role of the MSC secretome; for instance, Pendse et al. reported that MSC-derived microvesicles and exosomes can differentially regulate HSPC proliferation depending on whether they act in concert or independently [38]. Consistent with this, other studies evaluating extracellular vesicles and the MSC secretome have demonstrated growth-inhibitory effects or cell cycle arrest at the G0/G1 phase in hematopoietic models [39]. Taken together, this body of evidence indicates that the functional impact of the MSC secretome on hematopoiesis is highly context-dependent, varying with species, isolation methods, vesicular subsets, and pathological stressors such as fungal infection.

The Notch signaling pathway plays a central role in the regulation of HSPC self-renewal and maintenance, largely through interactions with MSCs [25,40]. In this study, the relative expression of *NOTCH1*, *NOTCH2*, and *JAGGED1* indicates that infection of MSCs with *H. capsulatum* differentially modulates this pathway through paracrine mechanisms. This result contrasts with the findings reported by Kim et al., who observed upregulation of Notch1 and Notch2 expression in HSPCs co-cultured directly with MSCs, compared to HSPC monocultures, demonstrating that MSCs modulate this signaling pathway. Furthermore, these authors reported that Notch2 activation helps HSPCs resist apoptosis, a correlation we could not establish in our study, as differential Notch2 expression alone did not fully account for our apoptotic outcomes [41]. The significance of this pathway is further underscored by the previous study demonstrating that Notch activation correlates with cell cycle regulation and hematopoietic recovery under stress conditions [41]. Although one study found that ectopic expression of Notch1 enhanced HSPC proliferation and survival [42], we did not observe a similar effect, which may be attributable to the fact that our approach measures mRNA expression levels rather than protein translation or the direct functional activation of the signaling pathway.

In general, the decreased expression of *NOTCH2* and *JAGGED1* under conditioned-medium conditions compared with co-culture conditions highlights that physical contact is not required to sustain their expression. In addition, the upregulation of *NOTCH1* in HSPCs exposed to conditioned medium from infected MSCs suggests a context-dependent regulation of Notch components.

Although no significant differences were observed for *JAGGED2*, a consistent trend toward decreased expression under conditioned medium was noted. Collectively, these findings suggest that *H. capsulatum* infection disrupts the balance of Notch signaling, with potential implications for hematopoietic homeostasis. Functionally, this dysregulation may be linked to the observed alterations in apoptosis, necrosis, and proliferation, thereby contributing to impaired hematopoietic support. Notably, Jagged1 has been shown to be critical for maintaining hematopoietic homeostasis and regenerative capacity, and its loss may compromise recovery following hematopoietic stress [43].

Importantly, the present study complements these observations by providing molecular evidence of infection-driven alterations in Notch signaling and apoptotic genes, including *NOTCH1*, *NOTCH2*, *JAGGED1*, and *BCL2*. Although Notch signaling and *BCL-2*-mediated survival are well-established regulators of hematopoietic cell fate and viability, their modulation in HSPCs following exposure to *H. capsulatum*-infected BM-MSCs has not been extensively characterized. Thus, our findings suggest that infection-induced changes in the stromal niche may influence HSPC homeostasis not only at the functional level but also through molecular pathways involved in cell survival, proliferation, and hematopoietic regulation.

These results provide insight into the hematological alterations observed in disseminated histoplasmosis, such as anemia and pancytopenia, by highlighting how infection-induced changes in the MSC secretome may shift the bone marrow niche from a homeostatic to a dysfunctional state [6,20]. Although the experimental model allows discrimination paracrine effects, it does not fully recapitulate the complexity of the in vivo niche, which includes additional cellular and molecular components regulating HSPCs [26].

### Limitations of the Study

While this study offers valuable insights into the stromal dysregulation underlying hematological complications in disseminated histoplasmosis, several methodological limitations remain. Primarily, the reliance on in vitro murine models using cells from C57BL/6 mice means these findings may not fully replicate the complex, dynamic microenvironment of human bone marrow in vivo. Furthermore, utilizing transwell co-cultures and conditioned media isolates paracrine mechanisms, potentially overlooking critical direct cell-to-cell contact interactions, extracellular matrix dynamics, and broader systemic immune responses present during live infection. Finally, the focus on a select set of molecular targets—specifically Notch signaling components (*NOTCH1*, *NOTCH2*, *JAGGED1*) and *BCL2*—leaves open questions regarding long-term temporal dynamics and the potential involvement of alternative apoptotic or immunomodulatory pathways. Bridging these findings to clinical application will require further validation using human cell models and in vivo disease studies.

## 5. Conclusions

Our findings reveal that *H. capsulatum* infection of MSCs disrupts the hematopoietic niche and alters the regulation of HSPCs by promoting apoptosis and necrosis, reducing proliferative capacity, and differentially modulating Notch signaling components. These alterations collectively indicate a shift from a supportive to a dysfunctional microenvironment, compromising the ability of HSPCs to maintain hematopoietic homeostasis. Importantly, this study highlights that the hematological manifestations observed in disseminated histoplasmosis may not only result from direct pathogen effects but also from infection-induced stromal dysregulation. Overall, these findings reinforce the importance of paracrine communication and may inform future strategies aimed at preserving or restoring bone marrow niche function during systemic infections.

## Figures and Tables

**Figure 1:**
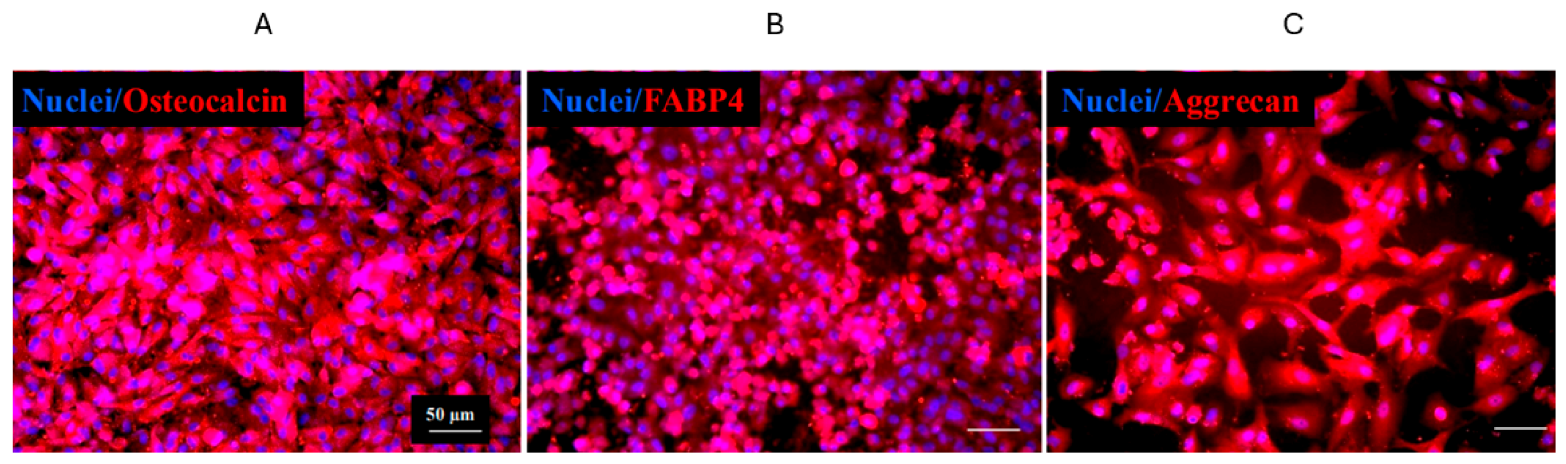
Morphological and phenotypic characterization of BM-MSCs. (**A**) Osteogenic differentiation, showing Hoechst nuclear staining and anti-osteocalcin immunofluorescence. (**B**) Adipogenic differentiation, showing Hoechst nuclear staining and anti-FABP4 immunofluorescence. (**C**) Chondrogenic differentiation, showing Hoechst nuclear staining and anti-aggrecan immunofluorescence.

**Figure 2:**
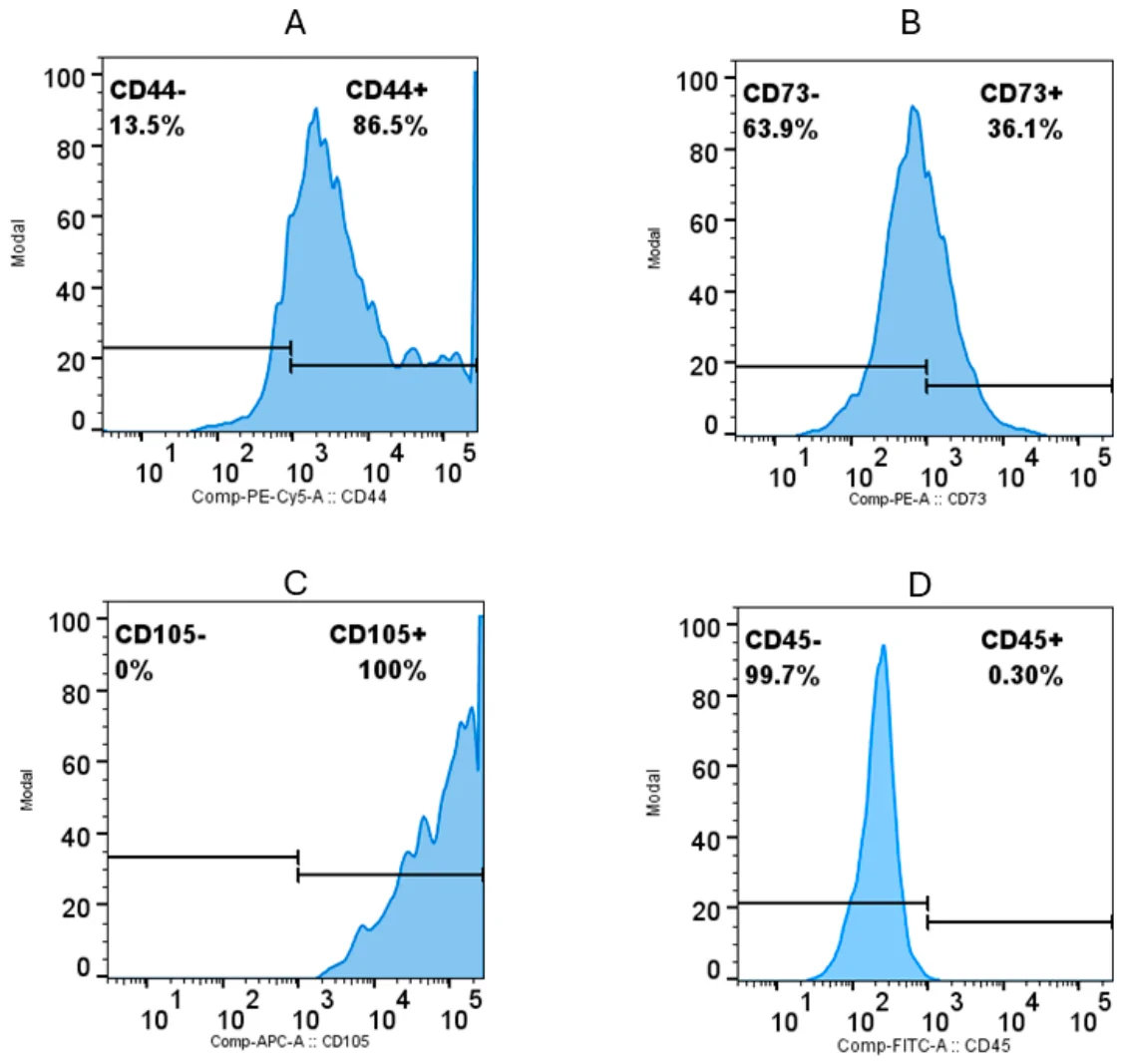
Flow cytometric characterization of BM-MSC surface markers. Representative histograms showing the expression of CD44 (**A**), CD73 (**B**), and CD105 (**C**), and the absence of CD45 expression (**D**).

**Figure 3:**
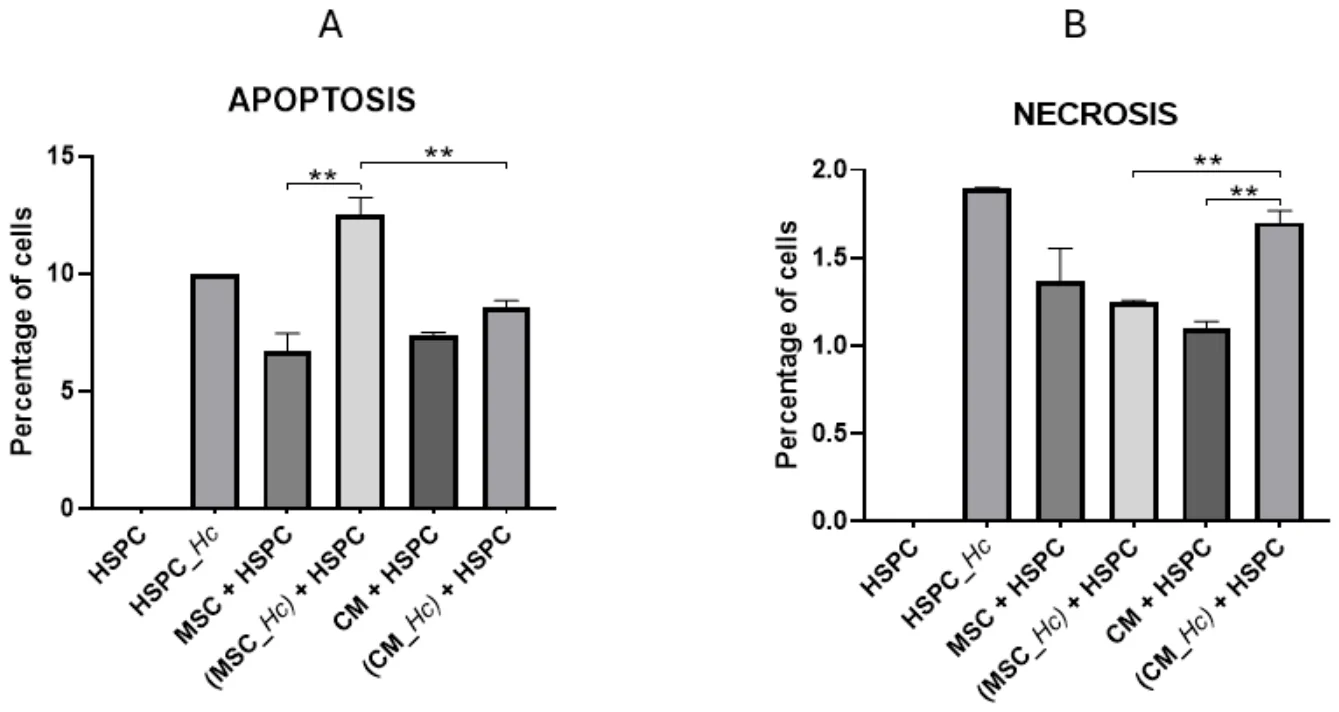
BM-MSCs infected with *H. capsulatum* increase apoptosis and necrosis in HSPCs. (**A**) Apoptosis, (**B**) Necrosis. The percentages represent the number of cells positive for FITC and propidium iodide. The data correspond to a representative experiment of three experiments performed in triplicate. ** *p* < 0.01. HSPCs, hematopoietic stem and progenitor cells; MSC, mesenchymal stromal cells; Hc, *Histoplasma capsulatum*; CM, conditioned medium.

**Figure 4:**
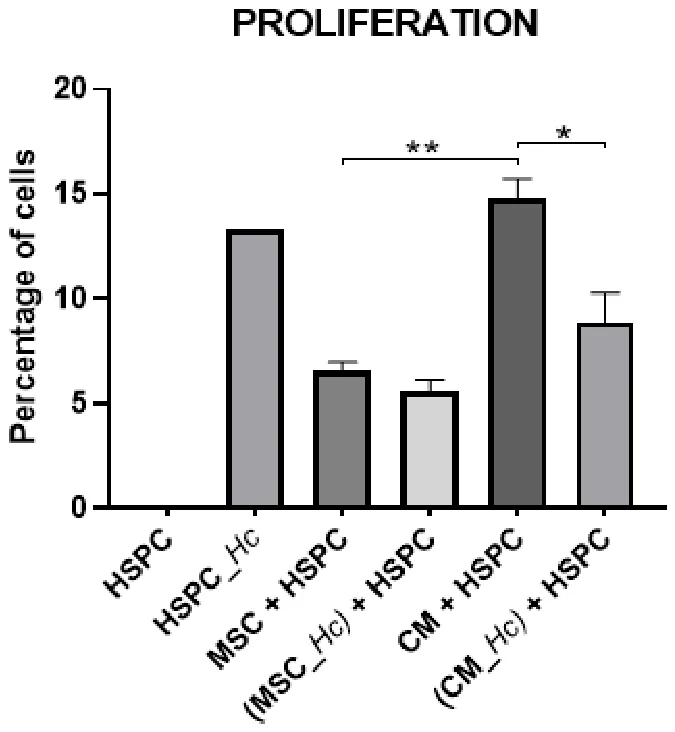
BM-MSCs infected with *H. capsulatum* decrease proliferation of HSPCs. Data represent the percentage of BrdU-positive cells and correspond to a representative experiment of three experiments in triplicate. * *p* < 0.05; ** *p* < 0.01. HSPC, hematopoietic stem and progenitor cells; MSC, mesenchymal stromal cells; Hc, *Histoplasma capsulatum*; CM, conditioned medium.

**Figure 5:**
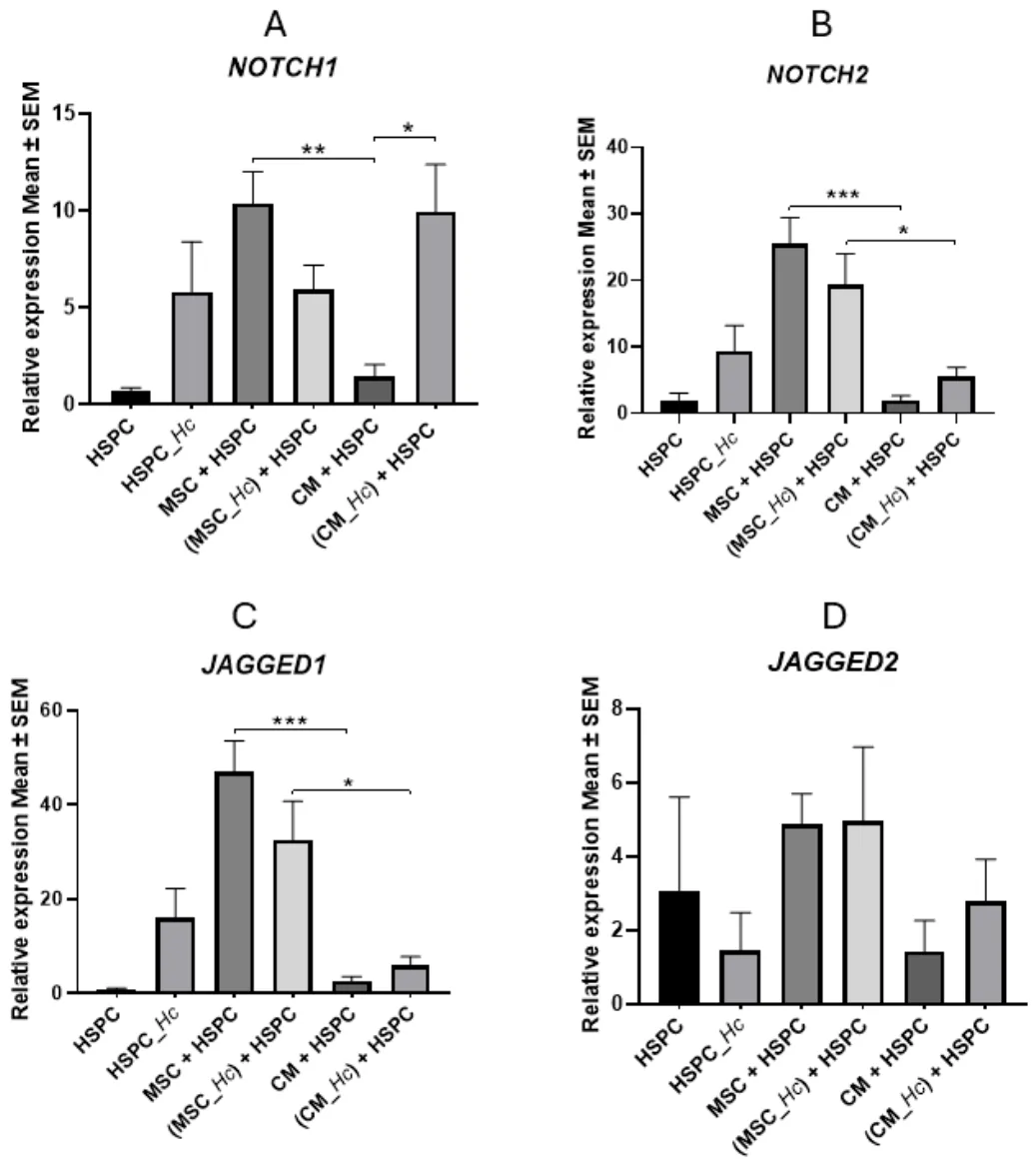
BM-MSC infected with *H. capsulatum* alters expression of signaling genes in HSPC. (**A**) *NOTCH1*, (**B**) *NOTCH2*, (**C**) *JAGGED1*, and (**D**) *JAGGED2*. * *p* < 0.05; ** *p* < 0.01; *** *p* < 0.001. HSPC, hematopoietic stem and progenitor cells; MSC, mesenchymal stromal cells; Hc, *Histoplasma capsulatum*; CM, conditioned medium.

**Figure 6:**
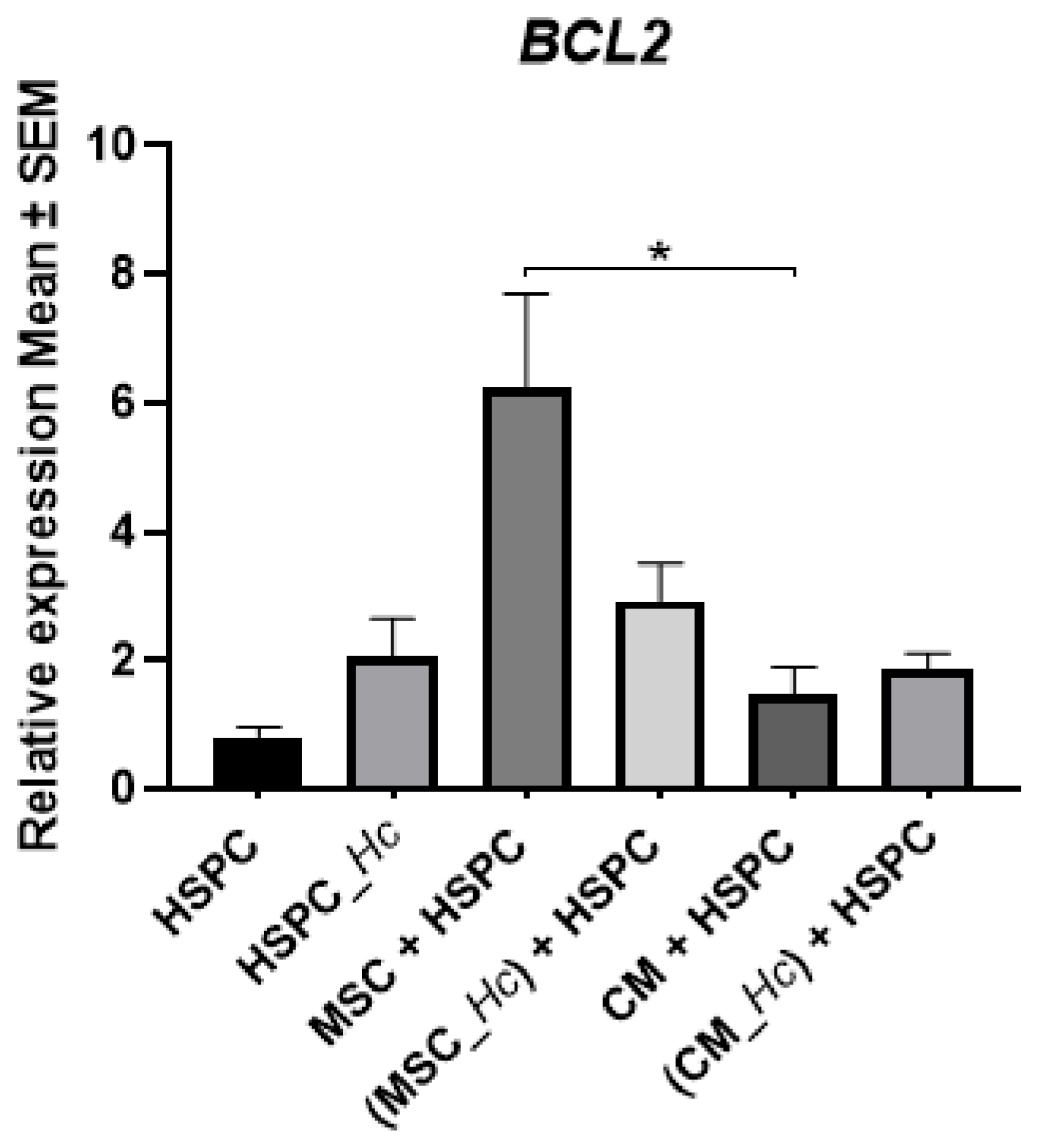
Effect of *H. capsulatum*-infected BM-MSCs on *BCL2* expression in HSPCs. Relative *BCL2* mRNA expression was assessed by RT-qPCR. * *p <* 0.05. HSPC, hematopoietic stem and progenitor cell; BM-MSC, bone-marrow-derived mesenchymal stromal cell; Hc, *H. capsulatum*; CM, conditioned medium.

**Table 1:** Sequences of primers for genes encoding proteins involved in the Notch signaling pathway and genes related to cell survival.

Gene	Forward (5′-3′)	Reverse (5′-3′)
*GAPDH*	CATGGCCTTCCGTGTTTCCTA	GCGGCACGTCAGATCCA
*NOTCH1*	ACATTCGCAGTAGAAGGAAGCC	GTGTGTGTCAATGGGTGGACT
*NOTCH2*	CGCCTTCCACTGTGAGTGTG	ACAGGTGAAGCCTCCGATCT
*JAGGED1*	CCGTTCTGGTCACAGGCATA	AACACAGGGATTGCCCACTT
*JAGGED2*	GCAGGTGGCACTGTAGTAGTT	TGATTGAGCGGGTGTCGC
*BCL-2*	ATCTCTGCGAAGTCACGACG	AGCCTGAGAGCAACCCAATG

## Data Availability

The original contributions presented in this study are included in the article. Further inquiries can be directed to the corresponding author.

## References

[1] Wagner U., Kaltenhäuser S., Pierer M., Wilke B., Arnold S., Häntzschel H. (2002). B lymphocytopenia in rheumatoid arthritis is associated with the DRB1 shared epitope and increased acute phase response. Arthritis Res. Ther..

[2] Weiss G., Goodnough L.T. (2005). Anemia of chronic disease. N. Engl. J. Med..

[3] LaMonte A.C., Paul M.E., Read J.S., Frederick M.M., Erdman D.D., Han L.L., Anderson L.J. (2004). Women and Infants Transmission Study Persistent parvovirus B19 infection without the development of chronic anemia in HIV-infected and -uninfected children: The Women and Infants Transmission Study. J. Infect. Dis..

[4] Utsinger P.D., Yount W.J. (1976). Lymphopenia in Sjögren’s syndrome with rheumatoid arthritis: Relationship to lymphocytotoxic antibodies, cryoglobulinemia, and impaired mitogen responsiveness. J. Rheumatol..

[5] Evatt B.L. (2005). HIV infection and thrombocytopenia. Curr. Hematol. Rep..

[6] Kobayashi H., Suda T., Takubo K. (2016). How hematopoietic stem/progenitors and their niche sense and respond to infectious stress. Exp. Hematol..

[7] Declercq W., Vanden Berghe T., Vandenabeele P. (2009). RIP kinases at the crossroads of cell death and survival. Cell.

[8] Di Paolo N.C., Doronin K., Baldwin L.K., Papayannopoulou T., Shayakhmetov D.M. (2013). The transcription factor IRF3 triggers “defensive suicide” necrosis in response to viral and bacterial pathogens. Cell Rep..

[9] Thapa R., Elfassy E., Olender L., Sharabi O., Gazit R. (2023). Rapid activation of hematopoietic stem cells. Stem Cell Res. Ther..

[10] Schürch C.M., Riether C., Ochsenbein A.F. (2014). Cytotoxic CD8+ T cells stimulate hematopoietic progenitors by promoting cytokine release from bone marrow mesenchymal stromal cells. Cell Stem Cell.

[11] Kovtonyuk L.V., Fritsch K., Feng X., Manz M.G., Takizawa H. (2016). Inflamm-aging of hematopoiesis, hematopoietic stem cells, and the bone marrow microenvironment. Front. Immunol..

[12] Rodríguez-Echeverri C., Gómez B.L., González Á. (2022). *Histoplasma capsulatum* activates hematopoietic stem cells and their progenitors through a mechanism dependent on TLR2, TLR4, and Dectin-1. J. Fungi.

[13] Megías J., Martínez A., Yáñez A., Goodridge H.S., Gozalbo D., Gil M.L. (2016). TLR2, TLR4 and Dectin-1 signalling in hematopoietic stem and progenitor cells determines the antifungal phenotype of the macrophages they produce. Microbes Infect..

[14] Welner R.S., Pelayo R., Nagai Y., Garrett K.P., Wuest T.R., Carr D.J., Borghesi L.A., Farrar M.A., Kincade P.W. (2008). Lymphoid precursors are directed to produce dendritic cells as a result of TLR9 ligation during herpes infection. Blood.

[15] Sioud M., Fløisand Y. (2007). TLR agonists induce the differentiation of human bone marrow CD34+ progenitors into CD11c+CD80/86+ DC capable of inducing a Th1-type response. Eur. J. Immunol..

[16] Ge Y., Wang J., Zhang H., Li J., Ye M., Jin X. (2022). Fate of hematopoietic stem cells determined by Notch1 signaling (review). Exp. Ther. Med..

[17] Wang W., Yu S., Myers J., Wang Y., Xin W.W., Albakri M., Xin A.W., Li M., Huang A.Y., Xin W. (2017). Notch2 blockade enhances hematopoietic stem cell mobilization and homing. Haematologica.

[18] Liu B., Zhou Y., Wu Q., Fu Y., Zhang X., Wang Z., Yi W., Wang H., Chen Z., Song Z. (2023). EVA1A regulates hematopoietic stem cell regeneration via ER-mitochondria mediated apoptosis. Cell Death Dis..

[19] Chen Z., Xia X., Yao M., Yang Y., Ao X., Zhang Z., Guo L., Xu X. (2024). The dual role of mesenchymal stem cells in apoptosis regulation. Cell Death Dis..

[20] Rodríguez-Echeverri C., Gómez B.L., González Á. (2023). *Histoplasma capsulatum* modulates the immune response, affects proliferation and differentiation, and induces apoptosis of mesenchymal stromal cells. Mycoses.

[21] World Health Organization (2022). WHO Fungal Priority Pathogens List to Guide Research, Development and Public Health Action.

[22] Emily M., Jessica S., John W. (2025). Histoplasmosis. Infect. Dis. Clin. N. Am..

[23] Deepe G.S., Blaser M.J., Cohen J.I., Holland S.M., Doi Y., Falsey A.R., Garret W.S., Marr K.A., Mitre E., Wilson E. (2026). *Histoplasma capsulatum* (Histoplasmosis). Mandell, Douglas, and Bennett’s Principles and Practice of Infectious Diseases.

[24] Schofield R. (1978). The relationship between the spleen colony-forming cell and the haemopoietic stem cell. Blood Cells.

[25] Kandarakov O., Belyavsky A., Semenova E. (2022). Bone marrow niches of hematopoietic stem and progenitor cells. Int. J. Mol. Sci..

[26] Mendelson A., Frenette P.S. (2014). Hematopoietic stem cell niche maintenance during homeostasis and regeneration. Nat. Med..

[27] Dominici M., Le Blanc K., Mueller I., Slaper-Cortenbach I.C.M., Marini F.C., Krause D.S., Deans R.J., Keating A., Prockop D.J., Horwitz E.M. (2006). Minimal criteria for defining multipotent mesenchymal stromal cells. The International Society for Cellular Therapy position statement. Cytotherapy.

[28] Baldridge M.T., King K.Y., Boles N.C., Weksberg D.C., Goodell M.A. (2010). Quiescent haematopoietic stem cells are activated by IFN-γ in response to chronic infection. Nature.

[29] Essers M.A.G., Offner S., Blanco-Bose W.E., Waibler Z., Kalinke U., Duchosal M.A., Trumpp A. (2009). IFNα activates dormant haematopoietic stem cells in vivo. Nature.

[30] Pietras E.M., Lakshminarasimhan R., Techner J.M., Fong S., Flach J., Binnewies M., Passegué E. (2014). Re-entry into quiescence protects hematopoietic stem cells from the killing effect of chronic exposure to type I interferons. J. Exp. Med..

[31] Yamada Y., Zheng Z., Jad A.K., Yamashita M. (2024). Lethal and sublethal effects of programmed cell death pathways on hematopoietic stem cells. Exp. Hematol..

[32] Nössing C., Ryan K.M. (2023). 50 years on and still very much alive: ‘Apoptosis: A basic biological phenomenon with wide-ranging implications in tissue kinetics’. Br. J. Cancer.

[33] Muddineni S.S.N.A., Katz-Even C., Zipin-Roitman A., Rasoulouniriana D., Singha Roy D., Aborgies R., Beider K., Raz Y., Solomon N., Hershkovitz G. (2026). Mesenchymal stromal cells modulate survival and regeneration of human hematopoietic stem cells via PGE2/cAMP signaling. Cell Death Dis..

[34] Zehnle P.M.A., Wu Y., Pommerening H., Erlacher M. (2022). Stayin’ alive: Bcl-2 proteins in the hematopoietic system. Exp. Hematol..

[35] Newton K., Strasser A., Kayagaki N., Dixit V.M. (2024). Cell death. Cell.

[36] Crippa S., Conti A., Vavassori V., Ferrari S., Beretta S., Rivis S., Bosotti R., Scala S., Pirroni S., Jofra-Hernandez R. (2023). Mesenchymal stromal cells improve the transplantation outcome of CRISPR-Cas9 gene-edited human HSPCs. Mol. Ther..

[37] Walenda T., Bokermann G., Ventura Ferreira M.S., Piroth D.M., Hieronymus T., Neuss S., Zenke M., Ho A.D., Müllerg A.M., Wagner W. (2011). Synergistic effects of growth factors and mesenchymal stromal cells for expansion of hematopoietic stem and progenitor cells. Exp. Hematol..

[38] Pendse S., Kale V., Vaidya A. (2025). Microvesicles secreted by CoCl2-primed mesenchymal stromal cells boost the functionality of hematopoietic stem cells cultured with them. Ann. Hematol..

[39] de Freitas F.A., Levy D., Reichert C.O., Sampaio-Silva J., Giglio P.N., de Pádua Covas Lage L.A., Demange M.K., Pereira J., Bydlowski S.P. (2024). Influence of human bone marrow mesenchymal stem cells secretome from acute myeloid leukemia patients on the proliferation and death of K562 and K562-Lucena leukemia cell lineages. Int. J. Mol. Sci..

[40] Vanderbeck A.N., Maillard I. (2019). Notch in the niche: New insights into the role of Notch signaling in the bone marrow. Haematologica.

[41] Kim A., Shim S., Kim M.J., Myung J.K., Park S. (2018). Mesenchymal stem cell-mediated Notch2 activation overcomes radiation-induced injury of the hematopoietic system. Sci. Rep..

[42] Kim J.H., Thimmulappa R.K., Kumar V., Cui W., Kumar S., Kombairaju P., Zhang H., Margolick J., Matsui W., Macvittie T. (2014). NRF2-mediated Notch pathway activation enhances hematopoietic reconstitution following myelosuppressive radiation. J. Clin. Investig..

[43] Lampreia F.P., Carmelo J.G., Anjos-Afonso F. (2017). Notch signaling in the regulation of hematopoietic stem cell. Curr. Stem Cell Rep..

